# Successful Sequential Use of the Antibody-Drug Conjugate Trastuzumab Deruxtecan After Progression on Sacituzumab Govitecan in a Recurrent Treatment-Resistant Ovarian Cancer Patient: A Case Report

**DOI:** 10.7759/cureus.94784

**Published:** 2025-10-17

**Authors:** Victoria M Ettorre, Michelle Greenman, Luca Palmieri, Cem Demirkiran, Alessandro D Santin

**Affiliations:** 1 Obstetrics and Gynaecology, Yale University, New Haven, USA; 2 Woman and Child Health and Public Health, Università Cattolica del Sacro Cuore, Fondazione Policlinico Universitario Agostino Gemelli IRCCS, Rome, ITA

**Keywords:** antibody-drug conjugate, her2, high grade serous ovarian cancer, sacituzumab-govitecan, trastuzumab-deruxtecan, trop2

## Abstract

Treatment of recurrent, platinum-resistant, high-grade serous ovarian cancer (HGSOC) remains a challenge. Novel treatment options based on antibody-drug conjugates (ADCs) are currently in clinical trials in platinum-resistant and recurrent ovarian cancer patients. Optimizing the sequential use of ADCs is an area of unmet need and of rising clinical importance. A 70-year-old with recurrent, metastatic, platinum-resistant HGSOC overexpressing TROP2 and HER2 experienced a significant response to the ADC trastuzumab deruxtecan (anti-HER2) after progression on sacituzumab govitecan (anti-TROP2). Following trastuzumab deruxtecan treatment, she experienced a remarkable and prolonged (i.e., over six months) clinical response with resolution of liver metastatic lesions and a decrease in the size of abdominal tumor masses, along with a decrease in CA-125. She has now completed eight cycles (i.e., over six months of treatment), and her disease continues to demonstrate a prolonged response to trastuzumab deruxtecan treatment. The ADC has been well tolerated at a dose of 5.4 mg/kg with no dose-limiting toxicity or need for dose reductions. HGSOC patients with chemotherapy and sacituzumab govitecan-resistant disease may experience durable responses with the sequential use of trastuzumab deruxtecan. ADCs with a similar cytotoxic payload but targeting a different antigen may represent effective treatment options in patients with HGSOC.

## Introduction

High-grade serous ovarian cancer (HGSOC) is biologically highly aggressive and is often diagnosed at an advanced stage (stage III or IV). It is additionally characterized by a high rate of recurrence, a low rate of cure, and an overall poor prognosis. The current standard of care for HGSOC includes complete cytoreductive surgery with platinum-based chemotherapy. Recurrence with this therapy is high; however, the five-year survival rate at 32% [1]. Recent developments in antibody-drug conjugates, or ADCs, have broadened the possibility of treatment from chemotherapy alone to biomarker-targeted delivery of chemotherapeutic medications. ADCs are created with a targeting mechanism for a specific surface biomarker, with an attached cleavable or uncleavable linker to a toxic payload (i.e., chemotherapy) [2]. A variety of ADCs targeting multiple biomarkers differentially expressed in human tumors compared to normal tissues are currently in different stages of clinical development. Two of the most appealing and clinically advanced biomarkers targeted by ADCs in gynecologic tumors are TROP2 and HER2.

Our research group has recently reported that 47% of ovarian tumors tested by immunohistochemistry (IHC) expressed moderate-to-strong TROP2 expression (i.e., 2+ and 3+) while an additional 40% demonstrated 1+ TROP2 staining (total 1+/2+/3+ TROP2 positivity in ovarian cancer = 87%) [3,4]. Accordingly, multiple ADCs targeting TROP2 are currently in Phase II and Phase III clinical trials in gynecologic cancer patients. For example, the clinical activity of sacituzumab govitecan (i.e., Trodelvy, Gilead Science Inc., Foster City, CA), an ADC targeting TROP2 currently approved by the FDA for the treatment of breast and bladder cancer, is currently being evaluated against platinum-resistant ovarian cancer patients in a Phase II Investigator-Initiated clinical trial at Yale University (NCT06028932). Encouraging preliminary clinical activity of anti-TROP2 ADCs such as sacituzumab govitecan, MK-2870 (sacituzumab tirumotecan), and datopotamab deruxtecan (Dato-DXd) ADC has recently been reported in endometrial as well as ovarian cancer patients [5]. In agreement with these results, a case report from our group has recently demonstrated the remarkable clinical activity of sacituzumab govitecan in a heavily pretreated platinum-resistant ovarian cancer patient [6]. Another ADC, fam-trastuzumab deruxtecan, targeting HER2, has recently been approved by the FDA for the agnostic treatment of patients with recurrent tumors demonstrating 3+ HER2 expression by IHC. The approval of fam-trastuzumab deruxtecan (i.e., Enhertu, Daiichi-Sankyo, Tokyo, Japan) was supported by the impressive clinical responses demonstrated in platinum-resistant ovarian cancer patients with 3+ HER2 positivity enrolled in the DESTINY-PanTumor02 trial [7].

All TROP2-directed ADCs currently either FDA approved (i.e., sacituzumab govitecan and datopotamab deruxtecan) or in Phase II/III clinical trials (i.e., sacituzumab tirumotecan and datopotamab deruxtecan) against gynecologic cancers, and the anti-HER2 ADC fam-trastuzumab-deruxtecan, share a similar toxic payload (i.e., a topoisomerase inhibitor). Importantly, because the same class of toxic payload is present in all these ADCs, patients with recurrent tumors are not allowed enrollment in TROP2/HER2 ADC clinical trials if they have received prior treatment with a topoisomerase I inhibitor-containing ADCs. However, this major eligibility exclusion criterion seems to be based more on speculation rather than scientific evidence of acquired resistance to topoisomerase inhibitor-chemotherapy, since minimal to no information is currently available in the literature about the optimal sequential use of ADCs in gynecologic cancer patients.

Here we present, to our knowledge for the first time, the case of a heavily pretreated patient with recurrent HGSOC, who demonstrated a remarkable and prolonged clinical response to fam-trastuzumab-deruxtecan (T-DXd) after progression on treatment with another ADC (sacituzumab govitecan) sharing a similar toxic payload (i.e., topoisomerase inhibitor).

## Case presentation

SD is a 70-year-old who was initially diagnosed with stage IIIC HGSOC in July 2013. At that time, she underwent a supracervical hysterectomy, bilateral salpingo-oophorectomy, and partial omentectomy. No lymphadenectomy was performed.

**Figure 1:**
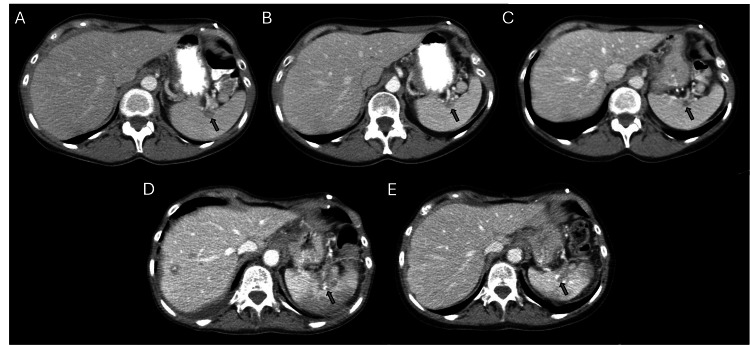
Evolution of the splenic implant during the patient’s disease course. Arrows in each CT A/P image indicate the splenic implant.

She then transferred her care from an outside institution to ours and was taken for a second-look surgery and intraperitoneal port placement in August 2013. Foundation Medicine testing was performed on her tumor and revealed a tumor mutational burden (TMB) of 1 mut/Mb, and alterations in ERBB2, CDK12, TP53, PRKCI, RAD21, SDHA, and TERC. Her first chemotherapy regimen was IV cisplatin-paclitaxel (cisplatin 60 mg/m^2^, paclitaxel 135 mg/m^2^) on day 1 and IP paclitaxel (60 mg/m^2^) on day 8. She completed a total of eight cycles by January 2014. Complications included admission for a partial SBO after her second cycle and pneumonia before her seventh cycle. Her CA125 from pre- to post-treatment had dropped from 2006 to 5.5 U/mL. Post-treatment CT showed minimal and non-specific omental disease, near complete resolution of lung consolidation (from pneumonia), and reduction in the size of the splenic implant (Figure 1A). A CT A/P performed three months later continued to show improvement (Figure 1B), and CA125 was stable at 5.7 U/mL. Her examinations and testing remained stable through 2015. In April 2016, her CA125 was noted to rise to 36.1 U/mL and then 51.9 U/mL, and a PET CT was obtained. It showed a 2.2 cm splenic implant. The patient (who was platinum-sensitive) received carboplatin, gemcitabine, and bevacizumab. She completed 12 cycles by March 2017. Due to thrombocytopenia before cycle 6, she had to delay her treatment by a week, and carboplatin was decreased to AUC 4. Her CT showed a decrease in the size of her splenic implant to 0.7 cm and a stable liver lesion (Figure 1C). Her CA125 was 9.4 U/mL after 12 cycles. 

She was then started on bevacizumab single-agent maintenance. After three months of maintenance, her CT showed stable disease (splenic implant 0.9 cm and liver implant 1.8 cm from 2.3 cm), and her CA125 was 8.9 U/mL. While on maintenance, she continued to have stable disease on CT, and her CA125 remained stable. After 12 cycles, the patient had repeat imaging with new induration in the subcutaneous area of a ventral hernia, and her CA125 had risen to 21.6 U/mL. The new area was unable to be biopsied, and she was then started on carboplatin single-agent chemotherapy in June 2018. After cycle 2, she had bone marrow toxicity and needed to have a dose reduction to AUC 3. After three cycles, she was reimaged and had stable disease with a CA125 of 13.4 U/mL. After extensive counseling, she was started on an oral PARP inhibitor (niraparib 200 mg daily) in October 2018. After three months of treatment, there was a significant improvement in peritoneal carcinomatosis. This stability continued while on 100 mg of niraparib. 

By March 2020, however, her CA125 had risen again to 41 U/mL, and her imaging had shown a new liver implant, which was ablated. When reimaged in September 2020, this area was decreased in size; however, there were new subcentimeter nodules in the liver with steadily increasing CA125 (now 145.3 U/mL). By October 2020, her CA125 was 239 U/mL. Given this increase in disease, IHC of her original tumor was performed, which showed HER2 3+, and she was started on trastuzumab single agent (8 mg/kg loading then 6 mg/kg every three weeks) in October 2020. After three cycles of trastuzumab, the patient had a CT scan “suggestive” of worsening peritoneal implants. Given this, she was scheduled for an MRI, which showed a decrease in the liver metastases that she previously exhibited, so she was continued on trastuzumab. During her first seven cycles, she was found to have the following CA125 pattern: 239 > 105 > 124 U/mL. Given this small rise, she was started on dose-dense paclitaxel (80 mg/m^2^ on days 1, 8, and 15) in addition to trastuzumab 4 mg/kg every two weeks. Her C1D15 paclitaxel was held for neutropenia. After three cycles of trastuzumab and paclitaxel, her CA125 was down to 80.7 U/mL. Her repeat CT showed stable liver disease, with the implant on the right liver edge resolved. The splenic hilar implant decreased from 2 to 1.8 cm. Later in 2021, after eight cycles, these scans were repeated with stable disease on CT. Her CA125 at the end of 2021 was 81.9 U/mL. CT was repeated in April 2022 with stable disease, even though CA125 was 141 U/mL. She was kept on her current regimen.

On her August 2022 scan, a 13 mm area on the liver was noted and suspicious for metastasis. She had an MRI performed, showing indeed worsening of the liver lesions and a CA125 of 244 U/mL. By this time, she had completed 17 cycles of paclitaxel and trastuzumab. With this progression of disease, she was started on a new regimen, cyclophosphamide 600 mg/m^2^ every three weeks with weekly paclitaxel in September 2022. CA125 at the time of initiation was 258 U/mL. 

CT and MRI in November 2022 showed stable disease but rising CA125 (highest 417 U/mL). After three cycles, her CA125 had decreased to 360 U/mL. In February 2023, she had a CT showing mild progression in her carcinomatosis, and CA125 had increased from 294 to 356 U/mL. Given this, she was started on ixabepilone (15 mg/m^2^/week) three weeks on and one week off with low-dose bevacizumab 5 mg/kg every other week.

By May 2023, she had a CT showing a mixed response; pelvic masses appeared decreased in size, but there were no changes to the implants noted in the upper abdomen and lymph nodes. Three additional cycles of ixabepilone and bevacizumab were completed, and CT after showed stable disease (August 2023). After her eighth cycle, she was seen for chest CT for shortness of breath and fatigue, which showed bilateral pleural effusions. She was to continue with ixabepilone and bevacizumab, with the addition of N-acetylcysteine (NAC) 600 mg daily or BID for inflammation. In November 2023, she had repeat imaging showing an increase in hepatic implants, an increase in lymphadenopathy, and worsening splenic infarcts (Figure 1D).

With limited options available, off-label use of sacituzumab govitecan (TROP2 ADC; 10 mg/kg days 1 and 8 of a 21-day cycle) was started in December 2023. After severe fatigue and leukopenia, she required filgrastim, and C1D8 was delayed. After four cycles of sacituzumab govitecan, she was noted to have a CA125 of 109 U/mL, down from 342 U/mL. CT scan confirmed improvement in the disease. She was seen again in April 2024 with an unchanged CT scan; however, there was an improvement in CA125 to 82.8 U/mL. She was continued on treatment. CT A/P was repeated in June 2024 with no changes, and CA125 was 110 U/mL. 

**Figure 2:**
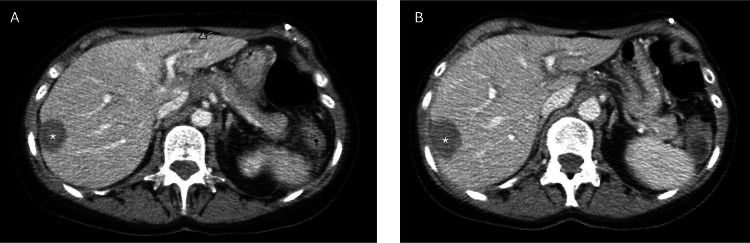
Resolution of liver metastasis after initiation of treatment with fam-trastuzumab-deruxtecan. CT A/P prior to treatment shows a sub-centimeter left liver lobe metastasis (A; arrow), now resolved after treatment with fam-trastuzumab-deruxtecan (B). Asterisk (*) indicates area of liver ablation treatment.

**Figure 3:**
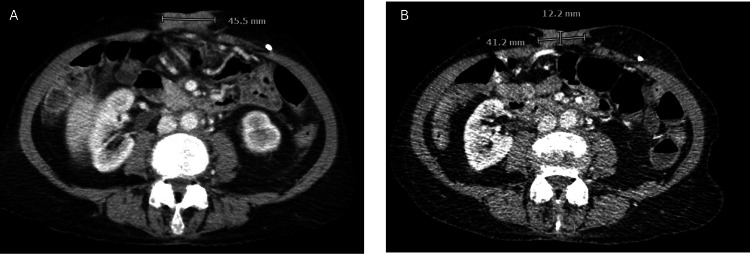
Decrease in size of a ventral abdominal wall implant after treatment with fam-trastuzumab-deruxtecan. After initiation of treatment, patient’s ventral abdominal mass decreased in largest dimension from 45.5 mm (A) to 41.2 mm (B).

**Figure 4:**
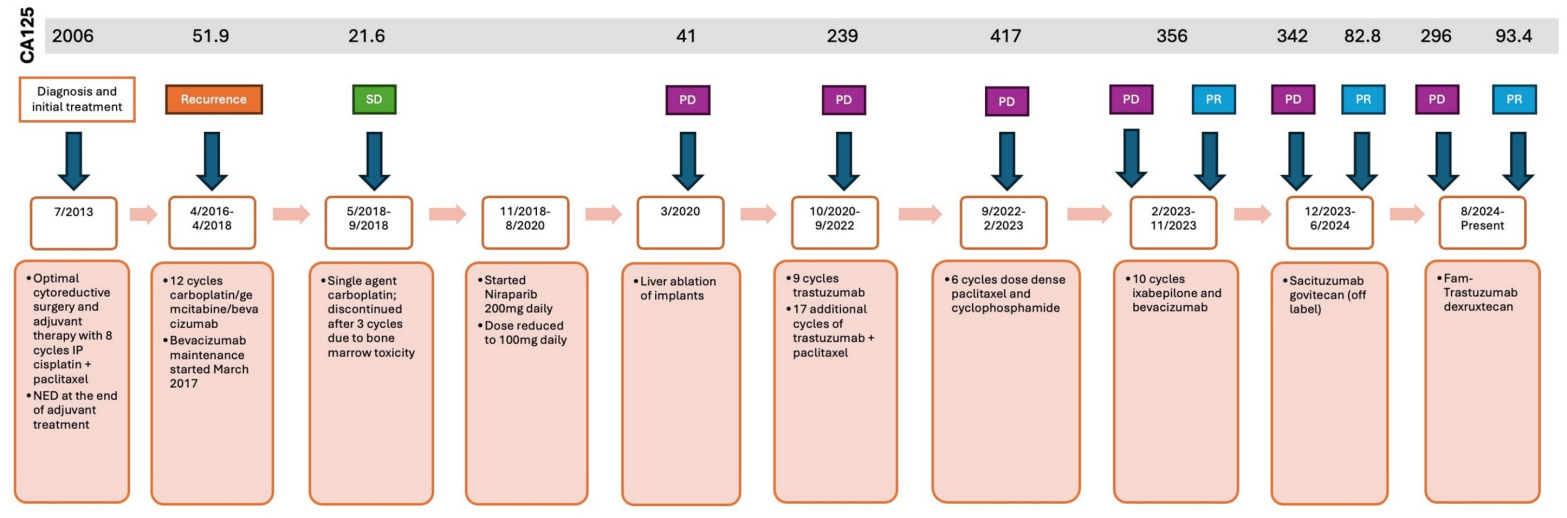
Timeline of patient’s disease course, CA125 values, and treatment response. SD = stable disease; PD = progression of disease; PR = partial response

After 12 cycles of sacituzumab govitecan, she had a repeat CT showing progression of disease; there was a soft tissue mass along the ventral abdomen (surgical scar mass), a decrease in the right hepatic lobe mass, and a new hypodensity in the left hepatic lobe. In August 2024, her treatment was changed to fam-trastuzumab-deruxtecan (T-DXd) 5.4 mg/kg day 1 of a 21-day cycle, as her tumor was HER2 3+, and this drug received recent FDA approval for HER2 3+ tumors. After three cycles, CT showed significant improvement in all metastatic disease sites, including the reduction of the splenic implant (Figure 1E) the resolution of liver metastases (Figure 2) and reduction in size of a large ventral tumor implant (Figure 3) with a concomitant decrease in CA125 biomarker (i.e., baseline CA125 = 296 U/mL, now down to 93.4 U/mL). After six cycles, CT scan and CA125 remained stable, confirming a durable clinical response. Patient is tolerating the T-DXd ADC well, and accordingly, is continuing treatment with fam-trastuzumab-deruxtecan at the time of the publication. Full clinical summary of treatments and responses can be seen in Figure 4. 

## Discussion

Here, we present a case of a patient with recurrent HGSOC, heavily pretreated with multiple lines of chemotherapy and progressing on sacituzumab govitecan, an ADC targeting TROP2, who demonstrated a prolonged response to fam-trastuzumab deruxtecan, an ADC targeting HER2 sharing a similar toxic payload (i.e., a topoisomerase I inhibitor) with sacituzumab govitecan.

To our knowledge, this case report represents the first successful demonstration of the sequential use of an ADC (fam-trastuzumab deruxtecan) after another ADC (sacituzumab govitecan) containing the same class of toxic payload in the treatment of chemotherapy-resistant HGSOC. During her clinical course, our patient was also treated with trastuzumab, a single agent (i.e., the unconjugated HER2-directed monoclonal antibody). Unfortunately, she quickly progressed on this monotherapy. While some of the mechanisms of action of ADC targeting HER2 are shared with the unconjugated (i.e., naked) trastuzumab mAb (e.g., the capability to induce strong NK-mediated antibody-dependent cell death (ADCC) of tumor cells expressing the HER2 target) [2], others, such as the ability to internalize a highly cytotoxic payload (i.e., chemotherapy) specifically into tumor cells expressing the specific biomarker, are unique to ADCs [8,9]. This additional mechanism of action may dramatically increase the clinical activity of the antibody [10,11]. Alternative therapeutic approaches such as immune checkpoint inhibitors, PARP inhibitors, and anti-angiogenic agents have shown variable efficacy in recurrent HGSOC, yet their benefit is often limited in patients with extensive prior treatment exposure and platinum resistance [1]. In this context, ADC-based strategies offer a rational and targeted approach for patients who have exhausted standard therapies. Consistent with this view, our group has previously demonstrated that ADCs targeting HER2 are remarkably more effective both in vitro and in vivo against gynecologic tumor models when compared to the unconjugated humanized monoclonal antibody targeting the same biomarker [12,13].

Since the FDA approval for the use of a TROP2-targeted ADC for gynecologic cancer patients is still pending, sacituzumab govitecan was approved for this ovarian cancer patient on a compassionate basis. Importantly, the second ADC used in this patient’s clinical care targeting the HER2 3+ positive tumor (i.e., T-DXd) is conjugated with a similar topoisomerase inhibitor toxic payload [14]. While the sequential use of ADCs has not yet been properly studied in gynecologic cancers, the remarkable and prolonged (i.e., over six months) clinical response detected in this HGSOC patient was somewhat unexpected considering the ineligibility of these patients for enrollment in clinical trial protocols if they have received prior treatment with a topoisomerase I inhibitor-containing ADC. In this regard, however, a retrospective review has been performed in metastatic breast cancer patients [15]. After looking at 193 patients, only 32 were noted to have received more than one ADC. Of these 32 patients, 17 were noted to have cross-resistance to the ADCs [15]. However, the majority of these patients had subsequent use of ADC with the same antibody target. Of interest, the authors of this study noted that their population exhibited more durable responses when subsequent ADC use targeted a different biomarker. Our case report in a heavily pretreated, chemotherapy resistant, HGSOC patient is in agreement with these retrospective review data in breast cancer patients, and suggest that, while further research is needed to elucidate the mechanism(s) of cross-resistance to the sequential use of ADC, the use of ADCs with a similar/identical cytotoxic payload but targeting a different antigen highly expressed on cancer cells may still represent a potentially effective treatment option in heavily pretreated patients with chemotherapy resistant ovarian cancer.

## Conclusions

HGSOC is an aggressive malignancy with a high risk of recurrence. Use of ADCs may represent a new tool of treatment for HGSOC patients; however, the optimal sequential use of ADCs for the treatment of platinum-resistant ovarian cancer remains an area of active research. Here we report the case of a heavily pretreated patient with recurrent HGSOC, who demonstrated a remarkable and prolonged clinical response to fam-trastuzumab-deruxtecan (T-DXd) after progression on treatment with another ADC (sacituzumab govitecan) sharing a similar toxic payload (i.e., topoisomerase inhibitor). Fam-trastuzumab-deruxtecan was well tolerated at a dose of 5.4 mg/kg with no dose-limiting toxicity or need for dose reductions.

This case highlights the potential clinical utility of sequential ADC therapy in patients with HGSOC who have exhausted standard treatments. HGSOC patients with chemotherapy and sacituzumab govitecan-resistant disease may experience durable responses with the sequential use of trastuzumab deruxtecan. ADCs delivering a similar cytotoxic payload but targeting a different antigen may provide effective responses and expand therapeutic options in heavily pretreated patients with HGSOC. Ongoing studies and prospective analyses of subsequent ADC use are warranted to confirm these findings and to better define optimal sequencing strategies for ADC responsiveness.
